# Supplementary material for: A single-walker approach for studying quasi-nonergodic systems
Source: Sci Rep. 2017 May 22;7:2242. doi: 10.1038/s41598-017-01704-5 (PMC5440385; doi:10.1038/s41598-017-01704-5)
Supplement: Supplementary file 1 — Supplementary information [file 41598_2017_1704_MOESM1_ESM.pdf]

A single-walker approach for studying quasi-nonergodic systems

Zilvinas Rimas[1,*] **and Sergei N. Taraskin**[2]

[1]Sidney Sussex College and Department of Chemistry, University of Cambridge, Cambridge, UK
[*]zr219@cam.ac.uk
[2]St. Catharine's College and Department of Chemistry, University of Cambridge, Cambridge, UK

SUPPLEMENTARY INFORMATION

S 1 Simulation parameters and correlation time

The updated JW simulations for Ising and lattice-gas models were performed with parameter S being in the range $S \in [2.2, 2.6] \times 10^9$ (the value of S depends on temperature and is measured in number of kinetic MC steps), and $R = 7 \times 10^4$. The geometric sequence of simulation temperatures, $T_{i+1}^{-1} = kT_i^{-1}$ (where $k = 1.007$), with $T_1^{-1} = 0.5$ and $T_1^{-1} = 0.6$ was used for the Ising and lattice-gas models, respectively.

In order to check if the length of the JW runs was enough to avoid correlations between the states in the time series of MC chain, we calculated the integrated correlation time τ_{corr}. This quantity describes the time scale, for which the MC simulated system still holds some memory of its previous states[1]. For a time sequence of fluid densities ρ_i, that the system had at the i-th time step, the value of τ_{corr} is defined as,

$$\tau_{\text{corr}} = \sum_{k=0}^{S} \frac{c_\rho(k)}{c_\rho(0)} , \tag{1}$$

where $c_\rho(k) = \langle \rho_i \rho_{i+k} \rangle - \langle \rho_i \rangle^2$ is the time autocorrelation function of fluid density with separation interval k. The integrated correlation time for fluid density ρ, measured in the number of kinetic MC steps, was obtained for the JW simulations of the aerogel sample at $\mu = -4.145$ and is shown in Fig. S1. The peak in τ_{corr} at $\beta \simeq 0.9$ marks the critical region, when the multiple metastable states were explored (See Fig. 3b in the main text) causing large density fluctuations and slow relaxation. The simulation time scale S exceeds the integrated correlation times τ_{corr} by several orders of magnitude for any range of temperatures.

All simulations have been performed using one core of Intel Xeon X5680 CPU unit. The maximum amount of RAM required by the JW simulation was below 20GB. Each stage corresponding to a temperature in $\{T_\alpha\}$ ran for approximately 20 minutes.

S 2 Lattice-gas models

S 2.1 Lattice-gas Hamiltonian

Within the lattice-gas model, the sorption of fluid in porous media, can be described by the following Hamiltonian[2,3],

$$\mathscr{H} = -w_{\text{ff}} \sum_{\langle ij \rangle} \tau_i \tau_j \eta_i \eta_j - w_{\text{mf}} \sum_{\langle ij \rangle} [\tau_i \eta_i (1 - \eta_j) + \tau_j \eta_j (1 - \eta_i)] , \tag{2}$$

where w_{ff} and w_{mf} are the parameters representing fluid-fluid and matrix-fluid interaction strength, respectively, and the sums are taken over all distinct pairs of the nearest-neighbour sites. The energy scale is defined by setting up $w_{\text{ff}} = 1$. The only parameter of the model, wettability $y = w_{\text{mf}}/w_{\text{ff}}$, depends on the material of the matrix as well as the type of fluid considered. Following Ref.[4], the value of the wettability is set to $y = 2$, which results in reasonable agreement between experimental and simulation results. For $y = 1/2$, the lattice-gas model described by Hamiltonian (2) can be mapped onto spatially correlated site diluted Ising model[2,5] but, in general case, onto random-field Ising model[6] with spatial correlations imposed by matrix sites. In case of no matrix, i.e. $\phi = 1$, the model describes condensation in bulk, which can be mapped onto the Ising model used as a test in the main text.

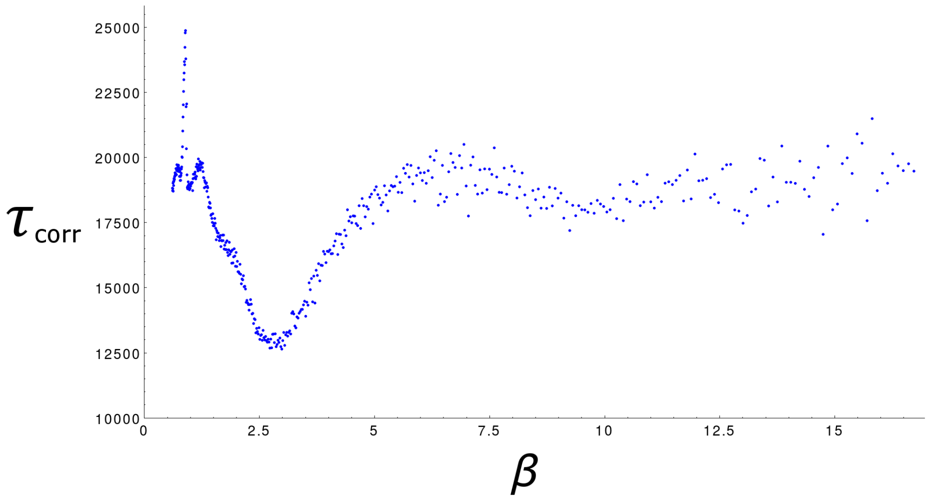

Figure S1. Integrated correlation time τ_{corr} measured in kinetic MC steps (vertical axis) for a range of inverse temperatures, β, obtained during the JW simulations of aerogel sample at $\mu = -4.145$.

S 2.2 Toy model

In order to test the updated JW algorithm we studied sorption of fluid in a bulk system (without matrix sites) modelled by small bcc lattice consisting of $2 \times 2 \times 3$ (total number of sites, $N = 2 \times 12 = 24$) primitive unit cells with periodic boundary conditions. For a system of such size it is possible to calculate numerically the grand partition function and other thermodynamic quantities exactly. This can be done in the following way.

In order to calculate exactly any observable of interest in equilibrium at some particular values of μ and β, one needs to sum over all possible microstates of the system. This task is often very computationally expensive (with some exceptions when the partition function is known analytically[7]), since the observable can be a function of all the sites. However, it is possible to simplify the procedure significantly by choosing a particular path over the state space, which ensures that each consecutive configuration along such a path differs from the previous one only at a single site. This way one can store the value of the observable at the previous configuration and alter it according to the change at a single site that has been either filled with or emptied of the fluid. This way we are able to reduce calculation of the observable for each microstate to a constant time operation (as oppose to being dependent in some higher degree on the system size).

For a system of N sites which of them can be in two states (empty or filled by fluid), the state of the entire system can be characterised by N-digit binary number, that we denominate as microstate signature. Gray code (also known as reflected binary code) is a method to alter the digits of a binary number in such a way that all possible numbers from 0 to 2^{N-1} are obtained only by switching one digit at a time. For example, Gray code for 2-, 3- and 4-bit binary numbers is given as follows,

2-bit	3-bit	4-bit
00	000	0000
01	001	0001
11	011	0011
10	010	0010
	110	0110
	111	0111
	101	0101
	100	0100
		1100
		1101
		1111
		1010
		1011
		1001
		1000

Employing efficient algorithm utilising bit-shift operations, it is possible to generate Gray code ”on-the-fly”, without storing

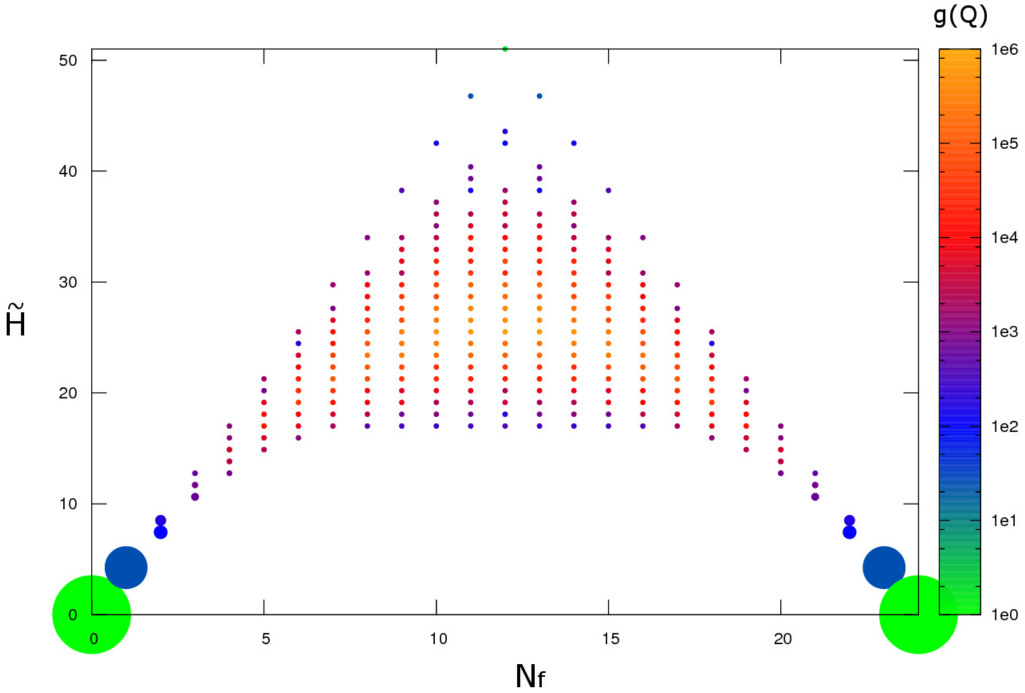

Figure S2. The state space, i.e. $\tilde{\mathscr{H}}$ *vs* number of sites occupied by fluid, N_f, of the $2 \times 2 \times 3$ bcc lattice containing 24 sites with periodic boundary conditions for $\mu = -4$ and $\beta = 1$. The colour of the data points reflects the values of the macrostate degeneracies, $g(Q)$, according to the colour scheme shown by the colour bar. The size of the data points proportional to the occupation probabilities of the macrostates.

any of the state variables for each microstate or even the microstate signatures themselves. The algorithm "knows" only the current state and the partial sums of the observables that we aim to obtain. This resolves memory problems which would be faced even for very small systems, if any microstate-level information was stored. However, exact enumeration methods fundamentally cannot escape the second computational limitation that is the processing power. Just to enumerate all the states, taking a single operation per state, for a system of size $N = 50$, and a processing unit of 1GHz the computation would take approximately 13 days. And even though it is possible to employ more powerful computers, since each added cell in the system doubles the computational power required, even the most powerful currently existing supercomputer would not be able to tackle system of size $N \gtrsim 10^2$, when employing such methodology.

By applying the state-enumeration procedure described above, we were able to calculate exactly the equilibrium occupation probabilities for all macrostates in the toy model. The results are presented in Fig. S2. Similarly to the zero-field Ising model (see Fig. 1 (a) in the main text), the most degenerate states are concentrated in the middle of the state space (i.e. $\rho \simeq 1/2$) but if the temperature is low enough then the occupation probabilities are higher for the states in the "corners" characterised by low energies. Exactly this situation is shown in Fig. S2 when, for $\beta = 1$, the most probable states are either fully empty or fully occupied system.

The exact results for occupation probabilities in small models can serve for testing the MC algorithms[7–9]. We tested the updated JW algorithm by calculating the ergodicity measure, $\chi^2(\beta,S)$, given by the following expression[10],

$$\chi^2(\beta,S) = \sum_Q \left[P(Q(\rho,\tilde{\mathscr{H}}),S) - P_{\mathrm{exact}}(Q(\rho,\tilde{\mathscr{H}}))\right]^2 , \tag{3}$$

where $\rho = N_\mathrm{f}/N$, $P(Q(\rho,\tilde{\mathscr{H}}),S)$ is the probability to visit macrostate Q by JW for finite MC process of S kinetic MC steps and $P_{\mathrm{exact}}(Q(\rho,\tilde{\mathscr{H}}))$ is the exact thermodynamic value for such probability. The ergodicity measure calculated at different temperatures is shown in Fig. S3 both for updated JW and single-flip processes. The small values of $\chi^2(\beta,S)$ being characteristic of ergodic behaviour are seen for both single-flip and JW processes at high temperatures. However, the single-flip dynamics ceases to be ergodic for $\beta \gtrsim 2$ while the JW process still remains to be ergodic. The ergodicity measure depends on the simulation time, i.e. on S, and, in the ergodic regime, it decays $\propto S^{-1}$[11] as we checked for the JW process (not shown).

The inverse-time decay of ergodicity measures is a general feature following from "diffusive" nature of all contributions in correlation functions[11] when a system is in ergodic regime. In order to reveal such a dependence for our toy model, we used an

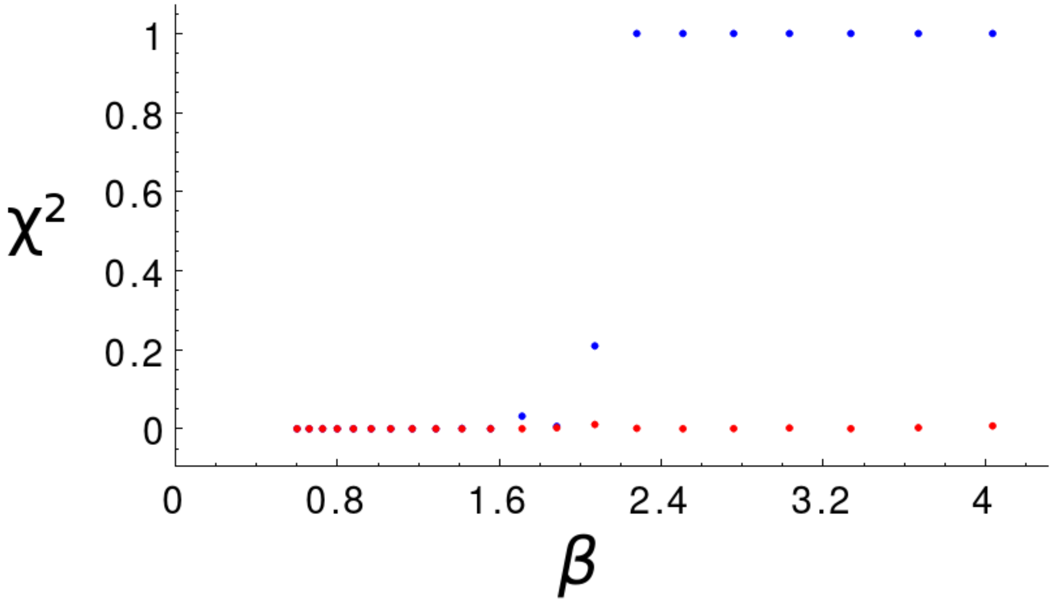

Figure S3. χ^2 *vs* β for the toy model system described in the text. The JW simulation and single-flip MC results are shown in red and blue respectively.

alternative and more general (not requiring exact solution) ergodicity metric d_k introduced in Ref.[11]. Let M be a number of independent executions of simulation that produce N-dimensional density sequences, $\{\rho_i^m(n)\}$, with m numbering the sequence, $m = 1,\ldots,M$, and n counting the lattice sites, $n = 1,\ldots,N$. The ergodicity metric, d_k, at the k-th MC step is defined as,

$$d_k = \frac{2}{M(M-1)} \sum_{i=2}^{M} \sum_{j}^{i-1} \sum_{n=1}^{N} \left[\overline{\rho_k^i}(n) - \overline{\rho_k^j}(n)\right]^2 , \tag{4}$$

where $\overline{\rho_k^i}(n)$ is the time averaged fluid density at the n-th lattice site up until k-th MC step during the i-th simulation, i.e.

$$\overline{\rho_k^i}(n) = \frac{1}{k+1} \sum_{\ell=0}^{k} \rho_\ell^i(n) . \tag{5}$$

We computed d_k metric for the toy model at different temperatures both for single-flip and JW processes (see Fig. S4). As follows from Fig. S4, the JW process is ergodic for all studied temperatures (cf. the slope of the thick blue line with the red dotted line representing the k^{-1} dependence). In contrast, the single-flip processes (see the thin solid lines in Fig. S4) loose ergodic behaviour at low temperatures.

It should be mentioned that some systems exhibit weak-ergodicity breaking[12] which is different from quasi-nonergodic behaviour studied here and for which a single-trajectory time averaging does not necessarily coincide with the ensemble averaging even in the limit of infinite observation time. This is a typical feature of diffusion models (thermal or athermal) with scale-free distribution of waiting times (with divergent mean sojourn time). In this situation, the limiting (infinite time) distribution for state occupation probabilities can differ from the Boltzmann distribution[13] and MC methods (including JW) might be useful for its sampling and studying non-ergodic dynamics.

S 2.3 Aerogel model

The structural models of silica aerogel[14–16] were generated by using the diffusion-limited cluster-cluster aggregation (DLCA) algorithm[17] run on bcc lattice consisting of $N_{\mathrm{tot}} = 2 \times L^3$ sites. Each realization of the modelled aerogel sample can be characterized by porosity, $\phi = {N_{\mathrm{tot}}}^{-1} \sum_i^{N_{\mathrm{tot}}} \eta_i$, which for real silica aerogels varies from 80% to 99.8%[16], and fractal dimension D. In this study, the DLCA algorithm was tuned to create cubic model samples with porosity $\phi = 95\%$ and $D \simeq 1.78$ that accurately reproduce the structural properties of the aerogels when compared to experimental neutron-scattering data[17,18]. In order to avoid fictitious "pinning" effects and simulate realistic desorption mechanism[4,19], an empty space slab ($\eta = 1$) of width equal to 4 lattice spacings, followed by a layer of enforced vapour ($\eta = 1$, $\tau = 0$) was attached to both sides of aerogel sample in z-direction. In the x- and y-directions periodic boundary conditions were imposed. The use of the bcc lattice allows to avoid lattice artefacts such as the faceted growth regime observed for the simple cubic lattices[4].

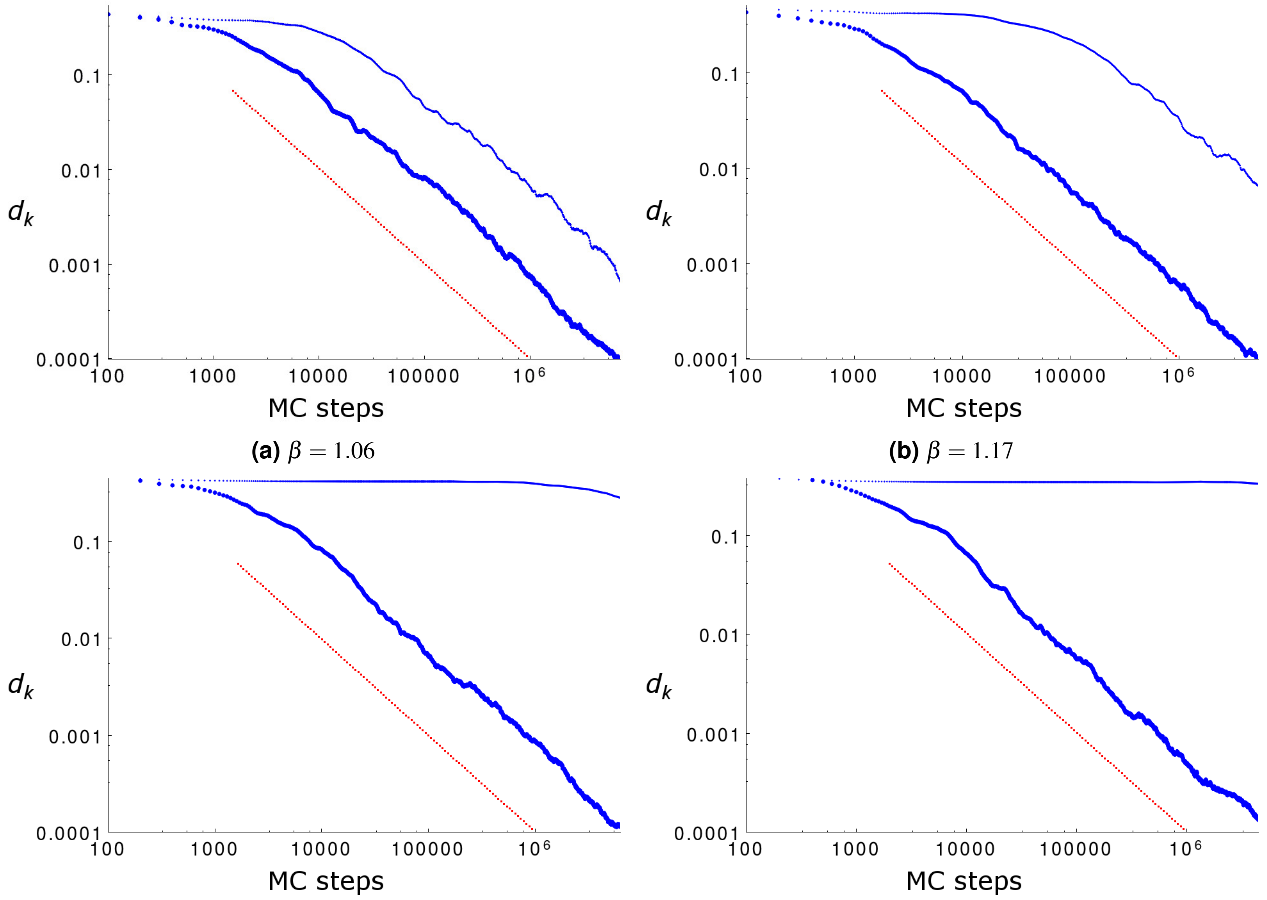

(a) $\beta = 1.06$

(b) $\beta = 1.17$

(c) $\beta = 1.56$

(d) $\beta = 1.71$

Figure S4. Ergodic measure d_k *vs* the number of kinetic MC steps k (the length of the simulation run), computed for the toy model at different temperatures β. The results for the JW and single-flip MC simulations are shown in thick and thin blue lines respectively. As a guide for an eye $d_k \propto k^{-1}$ line is displayed in red. $M = 100$.

S 2.4 Fluid configurations

Figs. S5A-S5D illustrate distributions of fluid in aerogel sample for the states characterised by different fluid densities marked by arrows A, B, C and D, respectively, in Fig.3 of the main text. The coloured surfaces show the boundaries between pore sites occupied and not occupied by fluid. The boundary layers are coloured according to their position in z-direction from blue (small z) to red (large z). The brown dots represent the matrix sites. Fig. S5A corresponds to the low-density minimum of $\tilde{\mathscr{H}}_{\min}(\rho)$, e.g. at $\rho \simeq 0.15$ for $\mu = -4.18$, when the fluid occupies the sites neighbouring the matrix sites. Fig. S5B describes the state corresponding to the high-density minimum of $\tilde{\mathscr{H}}_{\min}(\rho)$. For this state, the aerogel sample (for $5 \leq z \leq 20$) is filled by fluid, and only the added bulk layers (with $1 \leq z \leq 4$ and $21 \leq z \leq 25$) are left unoccupied, i.e fluid occupies the space between two approximately horizontal surfaces.

Figs. S5C-S5D illustrate the split, for intermediate values of μ around $\mu \simeq -4.145$, of high-density peak into two peaks which can be explained as follows. For this particular aerogel sample, the region of space with $z \simeq 5$ mainly populated by matrix sites is surrounded by a relatively empty space. This structural feature leads to existence of two fluid configurations of different density shown in Figs. S5C-S5D and corresponding to two most right minima in the grand potential landscape (solid line with open squares in Fig.3(b) of the main text). The fluid configuration of lower density with $\rho \simeq 0.35$ consists of pocket of pore sites occupied by fluid surrounding matrix sites near $z \simeq 5$ and separated by unoccupied pore space from the rest of aerogel sample occupied by fluid (see Fig. S5C). The fluid configuration of higher density with $\rho \simeq 0.45$ is similar to that with $\rho \simeq 0.35$ except that the empty pore sites separating the fluid pocket at $z \simeq 5$ from the rest of the fluid, in this case, are also occupied by the fluid (see Fig. S5D).

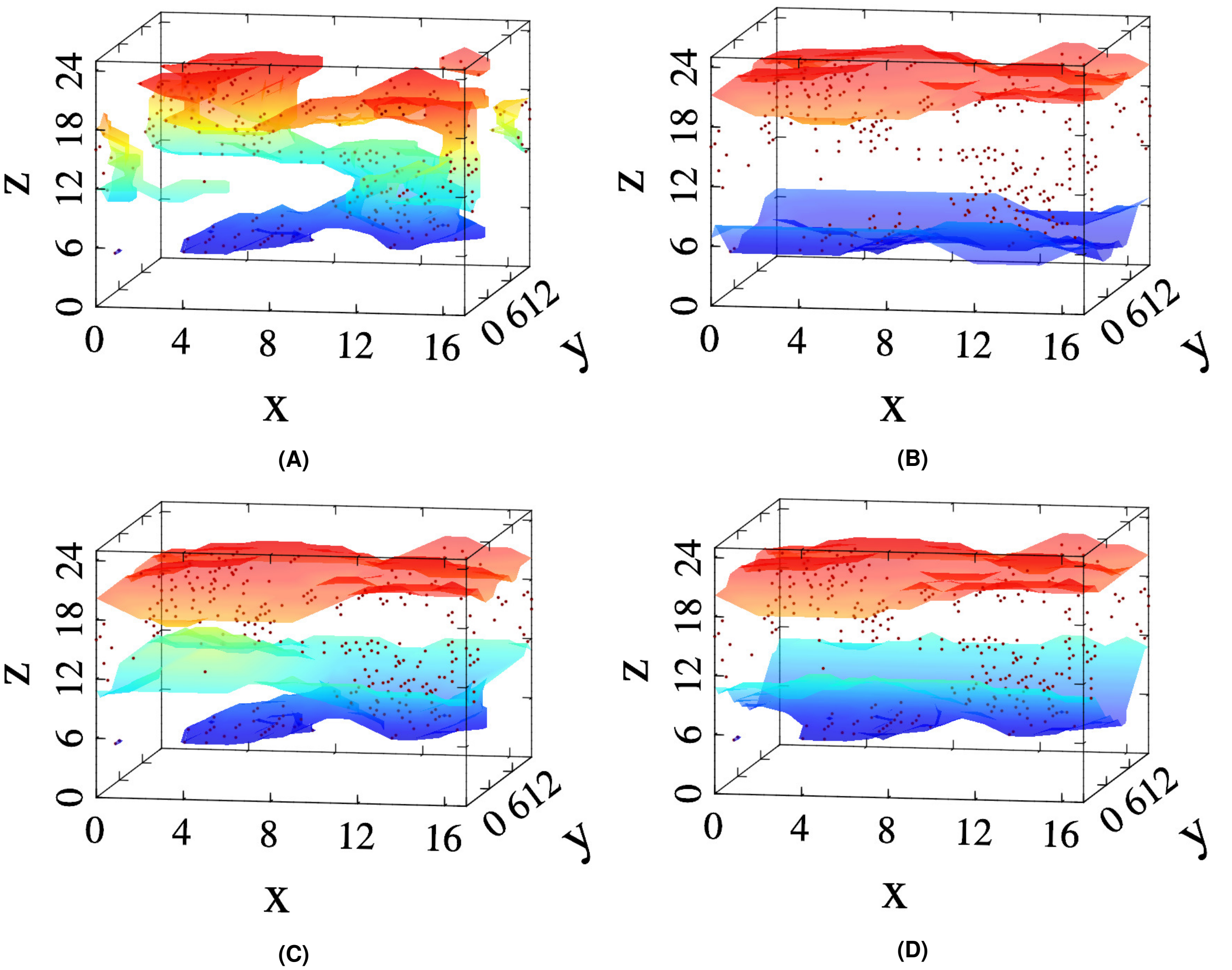

Figure S5. Fluid configurations for $\mu = -4.18$ (upper panel) and $\mu = -4.145$ (lower panel) labelled by A, B and C, D in Fig.3 of the main text, respectively.

S 3 High- and low-temperature boundaries

Here, we describe how the high-temperature $\tilde{\mathscr{H}}_{\max}(\bar{\rho})$ and low-temperature $\tilde{\mathscr{H}}_{\min}(\rho)$ boundaries for the state space displayed in Fig. 3 of the main text can be calculated.

At sufficiently high temperatures, $\beta^{-1} \gg 1$, each lattice site of the pore space is equally likely occupied by fluid, i.e. $\rho_i = \bar{\rho} = N_f/N$. The Hamiltonian of the system in this regime can be approximated by the mean-field value, $\mathscr{H}_{\max}(\bar{\rho})$, thus giving an estimate $\tilde{\mathscr{H}}_{\max}(\bar{\rho})$ for the high-temperature boundary at a fixed value of μ.

The approximate low-temperature limit for energy $\tilde{\mathscr{H}}_{\min}(\rho)$ is obtained through systematic exploration of the state space by random variation of β and μ while recording the lowest found value of $\mathscr{H}$ for each $\rho \in (0,1)$ according to the following algorithm.

(i) Run the standard single-flip MC simulation and record the energy $\mathscr{H}(\rho)$ of lowest energy state for each visited $\rho \in (0,1)$.

(ii) For every n-th MC step, randomly choose a value of β_{new} from the uniform distribution, $\beta_{\text{new}} \sim U(\beta_{\min}, \beta_{\max})$, and increment in chemical potential $\Delta\mu$ as either $\Delta\mu_{\text{step}}$ or $-\Delta\mu_{\text{step}}$, each with probability $p = 1/2$.

(iii) Set $\beta = \beta_{\text{new}}$ and $\mu = \mu_{\text{new}}$. Here, $\mu_{\text{new}} = \mu_{\max}$ if $\mu + \Delta\mu > \mu_{\max}$, $\mu_{\text{new}} = \mu_{\min}$ if $\mu + \Delta\mu < \mu_{\min}$ or $\mu_{\text{new}} = \mu + \Delta\mu$, otherwise.

(iv) Repeat (i)-(iii) until no new lowest value of $\mathscr{H}(\rho)$ at any ρ is found for m system (performing random walk in μ) traversals from $\mu_{\min}$ to $\mu_{\max}$ and back.

(v) Compute $\tilde{\mathscr{H}}_{\min}(\rho) = \mathscr{H}_{\min}(\rho) - \mu\rho$ for any μ of interest.

The system under this algorithm performs a bounded random walk in μ dimension. The bounds $\mu_{\min}$ and $\mu_{\max}$ are chosen such that the pore-space lattice sites of the system are either all empty or occupied by fluid at $\mu_{\min}$ and $\mu_{\max}$, respectively, ensuring that the entire range of $\rho \in [0,1]$ is explored. The step size, $\Delta\mu_{\text{step}}$, is chosen as small as possible, provided that the system still has enough time to diffuse from $\mu_{\min}$ to $\mu_{\max}$ and back multiple times throughout the simulation. The random variations in temperature provide an effective way to explore (search for and escape from) metastable states. The value of $\beta_{\min}^{-1}$ is chosen high enough, so that the system is insensitive to the local energy landscape ruggedness at such high temperature and explored states for any $\mu \in [\mu_{\min}, \mu_{\max}]$ span the wide range of ρ and $\tilde{\mathscr{H}}$ values. The low-temperature limit, $\beta_{\max}^{-1}$, the frequency, n^{-1}, of μ and β updates and the number of traversals m, are selected after the empirical exploration of algorithm's performance, ensuring that the further increase in $\beta_{\max}$, n and m does not improve the resultant values of $\mathscr{H}_{\min}(\rho)$. This procedure for finding $\mathscr{H}_{\min}(\rho)$ needs to be performed only once for a given aerogel model structure, since, for any particular value of μ, the estimate for $\tilde{\mathscr{H}}_{\min}(\rho)$ can be found by means of linear transformation given in step (v) of the algorithm.

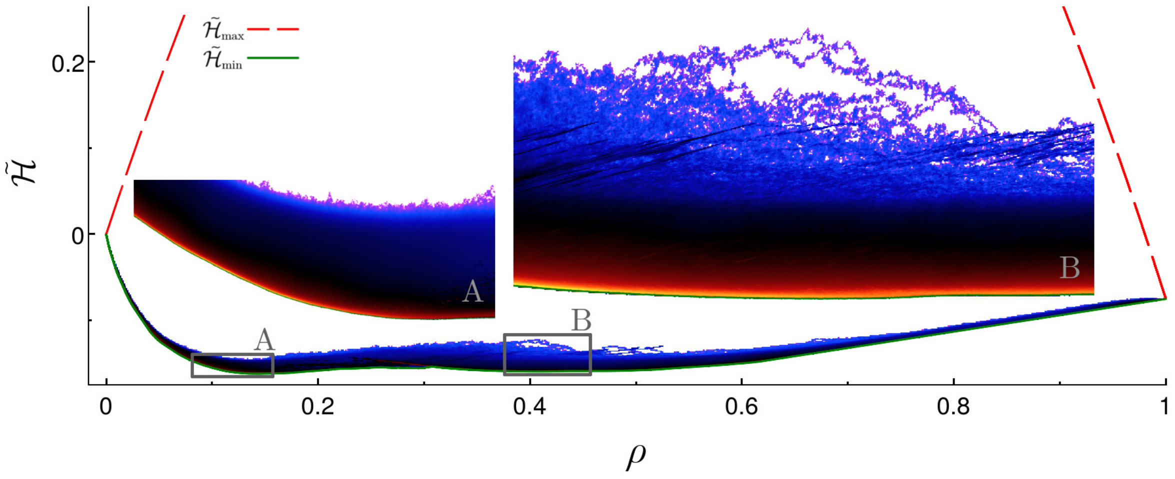

Figure S6. The colour map of the distribution of visits for a typical simulation run for search of the low-energy states using the suggested algorithm with the following set of parameters: $\mu_{\min} = -10$, $\mu_{\max} = 0$, $\Delta\mu_{\text{step}} = 0.001$, $\beta_{\min} = 2$, $\beta_{\max} = 20$, n in the range $[10^4, 10^5]$ depending on temperature, and $m = 20$. The insets show magnification of two regions (A and B) near the low-temperature boundary.

The algorithm was tested for a wide range of all parameter values in order to demonstrate that the low-energy boundary remains relatively unchanged if sufficiently high values of m are used. Fig. S6 illustrates the explored state-space using the same colour scheme as that in Figs. 1 and 3 for the distribution of visits after a characteristic execution of the algorithm for aerogel model analysed in the main text. The resultant distribution reaches maximum at $\tilde{\mathscr{H}}_{\min}(\rho)$ for fixed ρ which is evident from the insets in Fig. S6. The low-temperature boundaries in the state space shown in Fig. 3 of the main text were calculated with the same set of parameters as that used for obtaining the boundary presented in Fig. S6. It should be emphasised that $\tilde{\mathscr{H}}_{\min}(\rho)$ is not the strict state-space boundary, since in order to guarantee that no states exist with $\tilde{\mathscr{H}}(\rho) < \tilde{\mathscr{H}}_{\min}(\rho)$, an exhaustive search of the state space would be required, which is computationally infeasible for large systems. The boundary $\tilde{\mathscr{H}}_{\min}(\rho)$ is provided in Fig. 3 of the main text as a guideline to illustrate an approximate low-energy boundary of the state space for a particular realisation of aerogel at a fixed value of μ.

References

1. Landau, D. P. & Binder, K. *A guide to monte carlo simulations in statistical physics* (Cambridge University Press, 2005).
2. Kierlik, E., Rosinberg, M. L., Tarjus, G. & Pitard, E. Mean-spherical approximation for a lattice model of a fluid in a disordered matrix. *Mol. Phys.* **95**, 341–351 (1998).
3. Kierlik, E., Monson, P. A., Rosinberg, M. L., Sarkisov, L. & Tarjus, G. Capillary condensation in disordered porous materials: Hysteresis versus equilibrium behavior. *Phys. Rev. Lett.* **87**, 055701 (2001).
4. Detcheverry, F., Kierlik, E., Rosinberg, M. L. & Tarjus, G. Local mean-field study of capillary condensation in silica aerogels. *Phys. Rev. E* **68**, 061504 (2003).

5. Woo, H.-J. & Monson, P. A. Phase behavior and dynamics of fluids in mesoporous glasses. *Phys. Rev. E* **67**, 041207 (2003).

6. Sethna, J. P., Dahmen, K. A. & Perković, O. Random-Field Ising Models of Hysteresis. In Bertotti, G. & Mayergoyz, I. D. (eds.) *The science of hysteresis II* (Academic Press, New York, 2006).

7. Beale, P. D. Exact distribution of energies in the two-dimensional ising model. *Phys. Rev. Lett.* **76**, 78–81 (1996).

8. Papakonstantinou, T. & Malakis, A. Parallel tempering and 3d spin glass models. *J. Phys.: Conference Series* **487**, 012010 (2014).

9. Ren, Y., Eubank, S. & Nath, M. From network reliability to the ising model: A parallel scheme for estimating the joint density of states. *Phys. Rev. E* **94**, 042125 (2016).

10. Brown, S. & Head-Gordon, T. Cool walking: A new markov chain monte carlo sampling method. *J. Comp. Chem.* **24**, 68–76 (2003).

11. Neirotti, J. P., Freeman, D. L. & Doll, J. D. Approach to ergodicity in monte carlo simulations. *Phys. Rev. E* **62**, 7445–7461 (2000).

12. Metzler, R., Jeon, J.-H., Cherstvy, A. G. & Barkai, E. Anomalous diffusion models and their properties: non-stationarity, non-ergodicity, and ageing at the centenary of single particle tracking. *Phys. Chem. Chem. Phys.* **16**, 24128 (2014).

13. Bel, G. & Barkai, E. Weak ergodicity breaking in the continuous-time random walk. *Phys. Rev. Lett.* **94**, 240602 (2005).

14. Smirnov, B. M. Aerogels. *Sov. Phys. Uspekhi* **30**, 420–432 (1987).

15. Schaefer, D. W. & Keefer, K. D. Structure of random porous materials: Silica aerogel. *Phys. Rev. Lett.* **56**, 2199–2202 (1986).

16. Dorcheh, A. S. & Abbasi, M. Silica aerogel; synthesis, properties and characterization. *J. Mater. Proc. Tech.* **199**, 10–26 (2008).

17. Rahmani, A., Benoit, C., Jullien, R., Poussigue, G. & Sakout, A. Dynamical properties of a diffusion-limited cluster - cluster aggregation model. *J. Phys.: Cond. Matt.* **8**, 5555–5567 (1996).

18. Hasmy, A., Anglaret, E., Foret, M., Pelous, J. & Jullien, R. Small-angle neutron-scattering investigation of long-range correlations in silica aerogels: Simulations and experiments. *Phys. Rev. B* **50**, 6006–6016 (1994).

19. Rosinberg, M. L., Kierlik, E. & Tarjus, G. Percolation, depinning, and avalanches in capillary condensation of gases in disordered porous solids. *Europhys. Lett.* **62**, 377–383 (2003).
